# Sex-specific associations between telomere length and candidate miRNA expression in placenta

**DOI:** 10.1186/s12967-018-1627-z

**Published:** 2018-09-12

**Authors:** Maria Tsamou, Dries S. Martens, Bianca Cox, Narjes Madhloum, Karen Vrijens, Tim S. Nawrot

**Affiliations:** 1 Center for Environmental Sciences, Hasselt University, 3500 Hasselt, Belgium; 2 Environment & Health Unit, Department of Public Health, Leuven University (KU Leuven), 3000 Louvain, Belgium

**Keywords:** miRNA expression, Telomeres, Placenta, Sex

## Abstract

**Background:**

In the early-life environment, proper development of the placenta is essential for both fetal and maternal health. Telomere length at birth has been related to life expectancy. MicroRNAs (miRNAs) as potential epigenetic determinants of telomere length at birth have not been identified. In this study, we investigate whether placental miRNA expression is associated with placental telomere length at birth.

**Methods:**

We measured the expression of seven candidate miRNAs (miR-16-5p, -20a-5p, -21-5p, -34a-5p, 146a-5p, -210-3p and -222-3p) in placental tissue at birth in 203 mother-newborn (51.7% girls) pairs from the ENVIR*ON*AGE birth cohort. We selected miRNAs known to be involved in crucial cellular processes such as inflammation, oxidative stress, cellular senescence related to aging. Placental miRNA expression and relative average placental telomere length were measured using RT-qPCR.

**Results:**

Both before and after adjustment for potential covariates including newborn’s ethnicity, gestational age, paternal age, maternal smoking status, maternal educational status, parity, date of delivery and outdoor temperature during the 3rd trimester of pregnancy, placental miR-34a, miR-146a, miR-210 and miR-222 expression were significantly (*p *≤ *0.03*) and positively associated with placental relative telomere length in newborn girls. In newborn boys, only higher expression of placental miR-21 was weakly (*p *= *0.08*) associated with shorter placental telomere length. Significant miRNAs explain around 6–8% of the telomere length variance at birth.

**Conclusions:**

Placental miR-21, miR-34a, miR-146a, miR-210 and miR-222 exhibit sex-specific associations with telomere length in placenta. Our results indicate miRNA expression in placental tissue could be an important determinant in the process of aging starting from early life onwards.

**Electronic supplementary material:**

The online version of this article (10.1186/s12967-018-1627-z) contains supplementary material, which is available to authorized users.

## Background

Placental development is a key factor in fetal development and pregnancy outcomes. There is supporting evidence that adverse effects in the early-life environment can pose a risk of developing diseases in later-life, a concept known as “Developmental Origins of Health and Disease (DOHaD)” [1]. Fetal programming begins in the womb [2]. Epigenetic mechanisms, including microRNA (miRNA) expression modulation, may influence the fetal environment and programming [3] and subsequently the offspring’s health in adulthood. MiRNAs are short non-coding molecules (approximately 21 nucleotides) which target mRNA resulting in gene silencing by target degradation or translational repression at posttranscriptional level [4].

Telomeres (tandem TTAGGG repeats) protect the ends of chromosomes and the genetic information encoded by DNA. Telomeres shorten after each cellular division till a critical length known as “Hayflick limit” [5], as a result of the inability of DNA polymerase to completely replicate the lagging DNA strand [6]. Telomeres are maintained by the binding of a complex of proteins [telomeric repeat binding factor 1 and 2 (TRF1 and TRF2) proteins, the telomerase reverse transcriptase (TERT) protein itself and accessory factors EST1 and dyskerin] [7]. Telomere length is a complex trait, influenced by genetic factors (heritability of 60%) [8], host factors including sex [9–11], ethnicity [12], parental determinants including paternal age at birth [13], maternal education [14] and environmental factors such as psychosocial, behavioral and lifestyle factors [15–17]. Telomere attrition is one of the primary hallmarks in the aging process [18]. Inflammation and oxidative stress increase the rate of telomere attrition leading to telomere dysfunction [19]. Telomere length has been proposed as a biomarker of biological aging [20], is associated with many diseases [21–24] and has been implicated in pregnancy complications [25, 26]. Although at birth all newborns have the same chronological age (1 day old), they differ in their biological age as exemplified by telomere length [27]. Telomere length at birth has been associated with lifespan in Zebra finches [28] and has been shown to be predictive of adult telomere length [29].

Shorter placental telomere length was associated with maternal exposure to particulate air pollution [17] and maternal pre-pregnancy body mass index (BMI) [9], evidencing an important role for environmental stressors during pregnancy in the determination of placental telomere length.

Here, we studied seven miRNAs (miR-16, miR-20a, miR-21, miR-34a, miR-146a, miR-210 and miR-222), involved in fundamental biological processes such as inflammation (miR-21, miR-146a) [30, 31], cell cycle regulation (miR-16, miR-20a, miR-21, miR-34a, miR-210, miR-222) [32, 33], apoptosis (miR-16, miR-21) [34, 35], oxidative stress (miR-210) [36] and cellular senescence (miR-20a, miR-21, miR-34a, miR-146a, miR-210) [37]. We have previously shown that placental miRNA expression, including miR-20a, miR-21, miR-34a, miR-146a, miR-210 and miR-222, is linked to maternal exposure to air pollution (PM_2.5_ and NO_2_) [38] and/or pre-pregnancy BMI [39]. The cellular processes in which these miRNAs are involved, interact with the biology of healthy aging [18, 40].

It has been proposed that miRNAs may be implicated in the control of telomeres and subsequently in the process of aging [40]. Therefore, altered expression of miRNAs in early life and during aging could influence the expression of telomeric components and influence cellular lifespan. In the current study, we investigate the potential association of these candidate placental miRNA expression and biological aging at birth as reflected by placental telomere length.

## Methods

### Study population

Within the framework of the ongoing ENVIR*ON*AGE (ENVIRonmental influence ON AGEing in early life) birth cohort in the province Limburg in Belgium [41], 203 mother-newborn pairs were selected for this study, while twins were excluded. The mother-newborn pairs were recruited from March 2010 till January 2014 (from Friday to Monday). The study protocol was approved by the Ethical Committee of Hasselt University and South-East-Limburg Hospital (ZOL) in Genk (Belgium), and has been carried out according to the declaration of Helsinki. All participants agreed with written informed consent. The mothers had to be able to fill out questionnaires in Dutch. Overall, the rate of participation was around 61%. Participants had to provide detailed information about maternal age, education, occupation, smoking status, alcohol consumption, place of residence, use of medication and parity. Ethnicity of the newborn was classified based on the native country of the newborn’s grandparents: European-Caucasian (those with more than two European grandparents) and non-European (those with at least three non-European grandparents). Maternal smoking status was categorized into three groups: non-smokers (never smoked), past-smokers (quit smoking before pregnancy) and current-smokers (smoking during pregnancy). Lastly, maternal educational status was grouped as low (no diploma or primary school), middle (high school) or high (college or university degree). The maternal pre-pregnancy BMI (kg/m^2^) was recorded in the hospital at the first antenatal visit around week 7–9 of pregnancy. Maternal gestational weight gain (kg) was also retrieved from the hospital files. The mean daily outdoor temperature for the region of the study was provided by the Royal Meteorological Institute (Brussels, Belgium).

### Sample collection

Within 10 min of delivery, placental tissue was collected and samples were placed in RNA *later* immediately. After a 24 h incubation at 4 °C, samples were deep-frozen at − 20 °C the next day until use. Four standardized biopsies were taken at fixed locations across the middle point of the placenta around 4 cm distance from the umbilical cord, on the fetal side. Placental biopsies for DNA extraction were stored at − 80 °C.

### Selection of candidate miRNAs

**Fig. 1:**
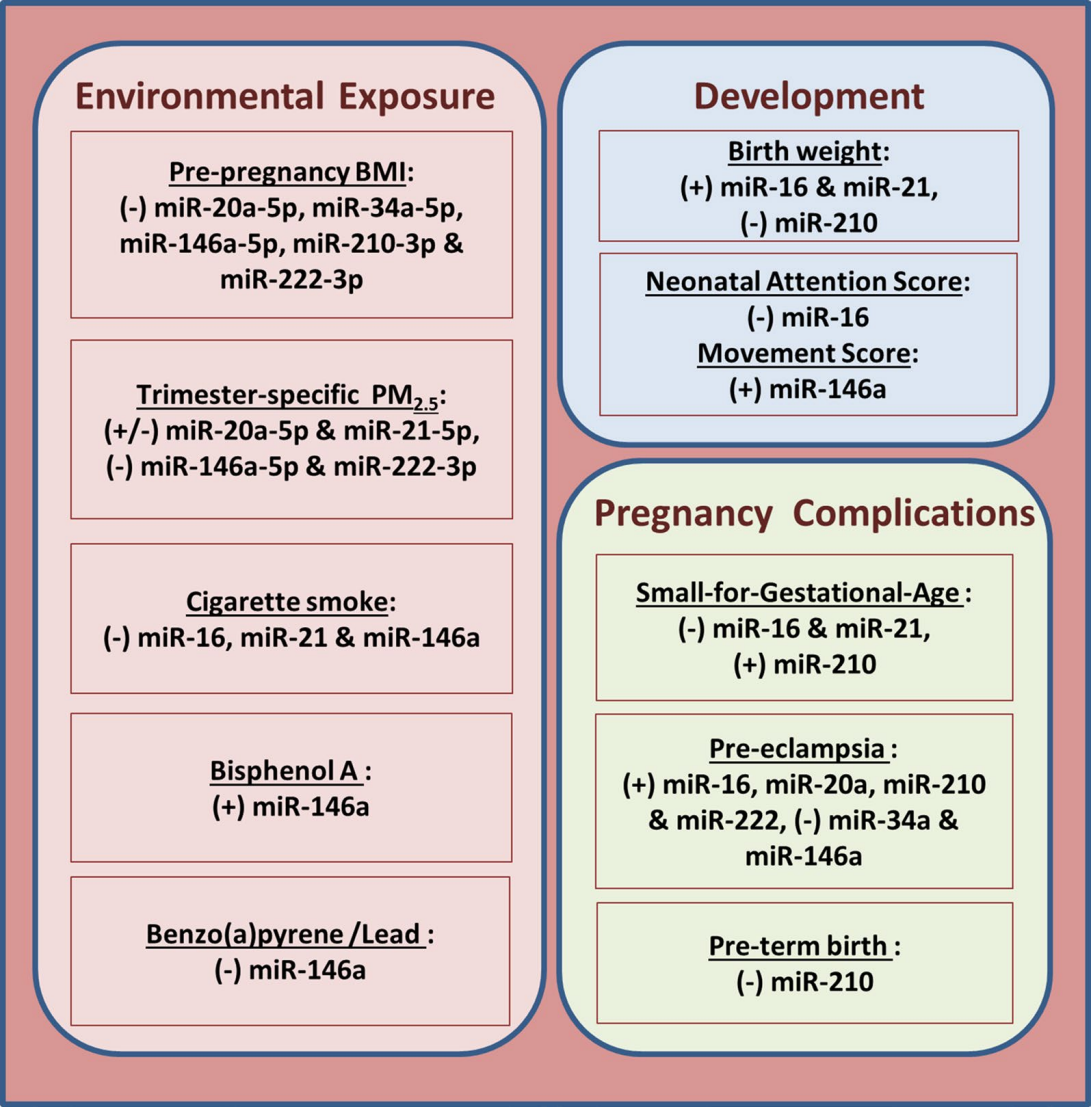
Associations of candidate miRNAs expressed in human placental tissue or cell lines with environmental exposures [38, 39, 42–44], fetal development [45–47] or pregnancy complications [46–52]

We chose seven miRNAs, including miR-16, miR-20a, miR-21, miR-34a, miR-146a, miR-210 and miR-222, which all have been previously reported to be involved in important cellular processes, including inflammation [30, 31], cell cycle regulation [32, 33], apoptosis [34, 35], oxidative stress [36] and cellular senescence [37]. These candidate miRNAs have been shown to be expressed in human placenta tissue or cell lines in association with environmental exposures [38, 39, 42–44], fetal developmental [45–47] or pathological conditions [46–52] during pregnancy (Fig. 1). We measured these miRNAs also in the context of two early life stressors: prenatal particulate matter pollution [38] and maternal pre-pregnancy BMI [39].

### RNA isolation and DNase treatment

Total RNA and miRNA were extracted from pooled placenta biopsies (6 mg tissue from each of the four available collected biopsies) using the miRNeasy mini kit (Qiagen, Venlo, The Netherlands) according to the manufacturer’s protocol. RNA quantity and purity was assessed by spectrophotometry (Nanodrop 1000, Isogen, Life Science, Belgium). DNase treatment was performed on extracted RNA samples according to the manufacturer’s instructions (Turbo DNA-free kit, Ambion, Life Technologies, Diegem, Belgium). Extracted RNA was stored at − 80 °C until further applications.

### Relative miRNA expression

RNA was reverse transcribed allowing miRNA specific cDNA synthesis of human miRNAs and small RNA controls, using TaqMan miRNA Reverse Transcription Kit (Applied Biosystems, Foster City, CA) and Megaplex stem-loop primer pool A (Applied Biosystems, Foster City, CA), according to the manufacturer’s protocol. After RNA reverse transcription into cDNA, the produced cDNA was stored at − 20 °C for further downstream measurements. For the PCR reactions, cDNA and Taqman miRNA assays (Applied Biosystems, Foster City, CA), were used on 7900HT Fast Real-Time PCR System (Applied Biosystems, Foster City, CA), according to the manufacturer’s protocol. Detailed methods have been described previously [38]. The following Taqman miRNA assays, including hsa-miR-16-5p (*assay ID:000391*), hsa-miR-20a-5p (*assay ID:000580*), hsa-miR-21-5p (*assay ID:000397*), hsa-miR-34a-5p (*assay ID:000426*), hsa-miR-146a-5p (*assay ID:000468*), hsa-miR-210-3p (*assay ID:000512*), hsa-miR-222-3p (*assay ID:002276*), and control U6 snRNA (*assay ID:001973*), were measured. The amplification efficiency of the miRNA assays was between 90 and 115%. Inter-run calibrators (IRCs) were used for minimizing the possible technical variation caused by the different runs of the same miRNA assays. Negative controls (non-template controls) were used in triplicate for each miRNA assay. SDS 2.3 software (Applied Biosystems, Foster City, CA) was used for the extraction of Cq values. Using qBase plus software (Biogazelle, Zwijnaarde, Belgium), the relative miRNA expression was calculated by calculated by 2^−ΔΔCq^ method. We measured all samples in triplicates and include only when ΔCq was smaller than 0.5.

### DNA isolation

Placental DNA was extracted from one placental tissue biopsy using the QIAamp DNA Mini Kit (Qiagen, Venlo, The Netherlands). We assessed DNA quantity and purity by a Nanodrop 1000 spectrophotometer (Isogen, Life Science, Belgium). DNA integrity was assessed by agarose gel-electrophoresis. As previously described [9], we assessed the within-placental average relative telomere length variation in 14 different placentas and for four different biopsies. As the within placental variation in telomere length was low, i.e. 11.7% we used only one biopsy (1–2 cm^3^) taken to the right of the main artery for placental telomere length assessment.

### Relative average placental telomere length

Telomere length was assessed by using a modified real-time PCR method [53]. In brief, for each sample in triplicate, the telomeric region was amplified with the use of telomere specific primers (telg and telc) and one single-copy gene was amplified (*36B4*) on a 7900HT Fast Real-Time PCR System (Applied Biosystems, Foster City, CA) in a 384-well format, specifications on PCR mixtures and cycling conditions are provided elsewhere [9]. After the amplification of the telomere specific region cycle thresholds were normalized relative to the cycle thresholds after the amplification of the single-copy gene using the qBase software (Biogazelle, Zwijnaarde, Belgium). Relative average placental telomere lengths were expressed as the ratio of telomere copy number to single copy gene number (T/S) relative to the average T/S ratio of the entire sample set. Reaction efficiency was assessed on each reaction plate (using a 6-point serial dilution of pooled placental DNA) and six different placental DNA samples were used as inter-run calibrators (IRCs) to account for inter-run variability. Negative controls (non-template controls) were also used in each run. We achieved coefficients of variation (CVs) of 0.55%, 0.35% and 7.1% for telomere runs, single-copy gene runs and T/S ratios, respectively.

### Prenatal particulate matter exposure

Particulate matter with a diameter ≤ 2.5 µm (PM_2.5_) was modelled by a interpolation method using hourly measured PM_2.5_ pollution data collected at fixed-site monitoring stations (n = 34) and land-cover data obtained from satellite images [54]. The model chain provides daily PM_2.5_ values on a dense, irregular receptor grid by using data both from the Belgian telemetric air-quality network and emissions from point sources and line sources. In the study region more than 80% of the temporal and spatial variability was explained with the model which also predicted children’s urinary carbon load [55]. During period of pregnancy, trimester-specific PM_2.5_ air pollution data were used based on three trimesters, which are defined as: trimester 1st (1–13 weeks), trimester 2nd (14–26 weeks) and trimester 3rd (27 weeks to delivery).

### Statistical analysis

SAS software (Version 9.4 SAS Institute, Cary, NC, USA) was used for the statistical analysis. The measured placental relative quantities of miRNA expressions and placental relative telomere lengths were both log-transformed (log10) because of their non-normal distribution. The associations of relative telomere length with placental relative miRNA expression at birth were assessed using multiple linear regression models, stratified by sex (n = 105 girls and n = 98 boys).

Placental miRNA expression has been previously shown to be influenced by maternal pre-pregnancy BMI (in some cases modified by gestational weight gain) [39], tobacco smoking [42], and gestational age [56]. Epigenetic mechanisms have been shown to be affected by temperature [39, 57], therefore adjustment for temperature was taken into account. In addition, placental telomere length has been previously associated with in utero environmental exposures such as pre-pregnancy BMI [9], air pollution [17] and tobacco smoking [58]. Newborn’s sex, paternal age [9], parity, gestational age [59] and race [60] were also shown to affect the placental telomere length.

In the *main analysis*, models were adjusted for following potential covariates: newborn’s ethnicity (European or non-European), gestational age (weeks), paternal age (years), maternal smoking status (never-smoker, past-smoker or current-smoker), maternal educational status (low, middle or high), parity (1, 2 or ≥ 3), date of delivery and outdoor temperature during the third trimester of pregnancy (categorized into quartiles, < 5.7 °C, ≥ 5.7 °C and < 9.4 °C, ≥ 9.4 °C and < 14.8 °C, ≥ 14.8 °C). The estimated effects were presented as percent change (95% CI) in placental relative telomere length for a doubling in relative miRNA expression. *P*-values were corrected for multiple testing (for the number of miRNAs studied) using the Benjamini–Hochberg false discovery rate (FDR) correction.

In a first *sensitivity analysis* (*Model 1*), we tested whether the ethnicity of the newborn affected these associations between placental miRNAs and telomere length, as racial differences in placental telomere length have been previously demonstrated [60]. The newborns with non-European origin were excluded from the analysis, resulting in a subpopulation consisting of 98 and 88 newborn girls and boys, respectively. In a second *sensitivity analysis* (*Model 2*), the main model was additionally corrected for potential confounders including maternal pre-pregnancy BMI (kg/m^2^) and gestational weight gain (kg). Finally, in a third *sensitivity analysis (Model 3)*, we additionally adjusted for trimester-specific PM_2.5_ air pollution during pregnancy, including all three trimester-specific PM_2.5_ windows in the model.

## Results

### General characteristics of the study population

The study population consisted of 203 mother-newborn pairs, detailed demographic characteristics are presented in Table 1. Mothers had an average (± SD) age of 29.5 (± 4.3) years and an average (± SD) maternal pre-pregnancy BMI of 24.1 (± 4.2) kg/m^2^. Most of the mothers never smoked cigarettes (69.9%) and had a high educational level (59.1%). Almost half (51.7%) of the newborns were girls and 91.6% had European-Caucasian ethnicity. The average (range) gestational age was 39.2  [35–41] weeks. The newborns had a mean birth weight of 3416 (± 445) g and the majority of them (49.3%) were the first child of their mother. The mean paternal age was 31.9 (± 5.5) years. Data of trimester-specific PM_2.5_ air pollution exposure for our study population are given in Additional file 1.

**Table 1:** Demographic characteristics of study population (n = 203)

Characteristics	Mean ± SD/frequency (%)		
	Both sexes (n = 203)	Boys (n = 98)	Girls (n = 105)
*Maternal*			
Age, years	29.5 ± 4.3	29.2 ± 4.3	29.8 ± 4.3
Pre-pregnancy BMI, kg/m^*2*^	24.1 ± 4.2	24.3 ± 4.1	23.9 ± 4.4
Gestational weight gain, kg	14.7 ± 6.1	14.7 ± 6.5	14.8 ± 5.3
Smoking status			
Never-smoker	142 (69.9)	67 (68.4)	75 (71.4)
Past-smoker	31 (15.3)	14 (14.3)	17 (16.2)
Current-smoker	30 (14.8)	17 (17.3)	13 (12.4)
Parity			
1	100 (49.3)	49 (50.0)	51 (48.6)
2	81 (39.9)	37 (37.8)	44 (41.9)
≥ 3	22 (10.8)	12 (12.2)	10 (9.5)
Education			
Low	21 (10.3)	11 (11.2)	10 (9.5)
Middle	62 (30.6)	35 (35.7)	27 (25.7)
High	120 (59.1)	52 (53.1)	68 (64.8)
*Paternal*			
Age, years	31.9 ± 5.5	31.7 ± 5.7	32.1 ± 5.3
*Newborn*			
Gestational age, weeks	39.2 ± 1.3	39.1 ± 1.4	39.3 ± 1.1
Birth weight, g	3416 ± 445	3469 ± 471	3366 ± 415
Ethnicity			
European-Caucasian	186 (91.6)	88 (89.8)	98 (93.3)
Non-European	17 (8.4)	10 (10.2)	7 (6.7)

### Association of placental relative miRNA expression with relative telomere length

The geometric mean of placental relative telomere length and relative miRNA expression (both log-10 transformed) are given in Table 2. Both before and after adjustment for potential covariates including newborn’s ethnicity, gestational age, paternal age, maternal smoking status, maternal educational status, parity, date of delivery and outdoor temperature during the 3rd trimester of pregnancy, higher placental relative expression of miR-34a, miR-146a, miR-210 and miR-222 was associated with longer placental relative telomere length, in newborn girls but not in boys (Table 3).

**Table 2:** Placental relative miRNA expression and relative telomere length (RTL) with (geometric) mean, interquartile ranger (IQR), 10th (P10) and 90th (P90) percentiles are given, in newborn girls and boys

	Mean	IQR	P10	P90	Mean	IQR	P10	P90
	Girls (n = 105)				Boys (n = 98)			
RTL	0.96	0.32	0.77	1.49	0.97	0.33	0.70	1.33
miR-16	0.99	1.11	0.24	4.87	0.95	1.85	0.23	3.94
miR-20a	1.08	1.52	0.27	4.20	0.94	1.40	0.19	5.72
miR-21	0.88	2.02	0.22	4.98	0.83	1.53	0.15	3.88
miR-34a	1.01	1.26	0.17	5.68	1.05	2.38	0.19	6.58
miR-146a	1.04	1.52	0.28	4.82	0.91	1.65	0.24	4.50
miR-210	0.96	1.86	0.17	6.88	0.95	2.05	0.15	5.59
miR-222	0.96	0.94	0.30	3.94	0.97	1.41	0.28	3.74

**Table 3:** Associations of placental relative telomere length (RTL) with placental relative miRNA expression, in newborn girls (n = 105) and boys (98)

miRNAs	Unadjusted analysis		Main analysis^a^		Sensitivity analysis					
					Model 1^b^		Model 2^c^		Model 3^d^	
	% Change in RTL (95% CI)	*p*-*value*	% Change in RTL (95% CI)	*p*-*value*	% Change in RTL (95% CI)	*p*-*value*	% Change in RTL (95% CI)	*p*-*value*	% Change in RTL (95% CI)	*p*-*value*
*Girls*										
miR-16	2.38 (− 0.15, 4.96)	0.068	1.93 (− 0.88, 4.82)	0.18	2.38 (− 0.53, 5.37)	0.11	2.02 − 0.86, 4.98)	0.17	2.42 (− 0.45, 5.38)	0.10
miR-20a	2.56 (− 0.13, 5.31)	0.065	2.14 (− 0.78, 5.14)	0.16	2.52 (− 0.47, 5.60)	0.10	2.28 (− 0.73, 5.38)	0.14	3.22 (0.14, 6.39)	0.044
miR-21	1.10 (− 1.04, 3.30)	0.32	0.78 (− 1.55, 3.17)	0.52	1.39 (− 1.06, 3.89)	0.27	0.82 (− 1.56, 3.26)	0.50	1.22 (− 1.18, 3.67)	0.33
miR-34a	2.60 (0.60, 4.64)	0.012*	2.84 (0.67, 5.05)	0.012*	3.05 (0.78, 5.37)	0.010*	2.96 (0.68, 5.29)	0.012*	3.22 (1.04, 5.45)	0.005*
miR-146a	3.57 (0.98, 6.22)	0.008*	3.44 (0.70, 6.26)	0.016*	3.48 (0.68, 6.37)	0.017*	3.55 (0.74, 6.44)	0.015*	3.95 (1.17, 6.81)	0.006*
miR-210	2.70 (0.67, 4.77)	0.010*	2.51 (0.28, 4.79)	0.030*	2.73 (0.40, 5.10)	0.024*	2.60 (0.31, 4.94)	0.028*	2.77 (0.53, 5.06)	0.017*
miR-222	4.05 (1.29, 6.89)	0.005*	3.61 (0.58, 6.74)	0.022*	3.99 (0.79, 7.30)	0.016*	3.74 (0.63, 6.94)	0.020*	4.73 (1.57, 7.99)	0.004*
*Boys*										
miR-16	0.09 (− 2.76, 3.04)	0.95	− 1.15 (− 3.99, 1.78)	0.44	− 1.85 (− 4.60, 0.98)	0.21	− 1.32 (− 4.16, 1.61)	0.37	− 1.43 (− 4.34, 1.56)	0.35
miR-20a	− 0.50 (− 3.27, 2.36)	0.73	− 0.55 (− 3.27, 2.26)	0.70	− 1.20 (− 3.84, 1.52)	0.39	− 0.73 (− 3.48, 2.09)	0.61	− 1.05 (− 3.86, 1.85)	0.48
miR-21	− 2.03 (− 4.47, 0.49)	0.12	− 2.20 (− 4.62, 0.28)	0.085	− 2.48 (− 4.78, − 0.12)	0.043	− 2.22 (− 4.64, 0.26)	0.082	− 2.44 (− 4.89, 0.08)	0.061
miR-34a	0.59 (− 1.83, 3.07)	0.64	− 0.93 (− 3.41, 1.61)	0.47	− 1.39 (− 3.82, 1.09)	0.27	− 1.10 (− 3.58, 1.45)	0.40	− 1.45 (− 3.95, 1.10)	0.27
miR-146a	− 0.23 (− 3.24, 2.88)	0.89	− 0.32 (− 3.37, 2.83)	0.84	− 0.58 (− 3.58, 2.51)	0.71	− 0.50 (− 3.56, 2.65)	0.75	− 1.14 (− 4.30, 2.12)	0.49
miR-210	− 0.08 (− 2.28, 2.17)	0.94	− 0.03 (− 2.26, 2.25)	0.98	− 0.89 (− 3.05, 1.32)	0.43	− 0.31 (− 2.57, 2.01)	0.79	− 0.38 (− 2.62, 1.92)	0.75
miR-222	0.40 (− 2.74, 3.64)	0.81	− 0.85 (− 3.91, 2.31)	0.60	− 1.08 (− 4.06, 1.99)	0.49	− 1.28 (− 4.37, 1.91)	0.43	− 1.97 (− 5.20, 1.38)	0.25

Estimated effects are presented with the obtained % change (95% CI) in placental RTL for a doubling in miRNA expression

*Remained significant after FDR correction (FDR ≤ 0.05)

^a^Main analysis was adjusted for newborn’s ethnicity, gestational age, paternal age, maternal smoking status, maternal educational status, parity, date of delivery and outdoor temperature during the 3rd trimester of pregnancy

^b^Excluding newborns with non-European ethnicity (girls: n = 98, boys: n = 88)

^c^Main analysis was additionally adjusted for pre-pregnancy BMI and gestational weight gain during pregnancy

^d^Main analysis was additionally adjusted for trimester-specific PM_2.5_ air pollution

In the *main analysis*, we performed a linear regression accounting for aforementioned covariates. In newborn girl, we found for each doubling in placental miRNA expression for respectively miR-34a, miR-146a, miR-210 and miR-222 a 2.84% (95% confidence interval [CI] 0.67, 5.05, *p *= *0.012*), a 3.44% (95% CI 0.70, 6.26, *p *= *0.016*), a 2.51% (95% CI 0.28, 4.79, *p *= *0.030*) and a 3.61% (95% CI 0.58, 6.74, *p *= *0.022*) longer telomeres at birth. In newborn boys, only miR-21 showed a trend towards significance in association with telomere length: for a doubling in placental miR-21 expression a shorter placental relative telomere length of 2.20% (95% CI − 4.62, 0.28, *p *= *0.085*).

In *Model 1* we excluded newborns with non-European origin (n = 17), slightly stronger effects were found in girls and boys. Further, in the *sensitivity analyses (Model 2 and 3)* no changes in significance levels and estimates were observed after additionally adjusting for maternal pre-pregnancy BMI and gestational weight gain or trimester-specific PM_2.5_ air pollution exposure during pregnancy, neither in girls nor boys (Table 3). The associations were not altered importantly after adjusting for multiple testing by FDR (Additional file 2).

## Discussion

Human aging is a complex process in which telomere length is involved in the deterioration of cellular function. We [9] and others [61] demonstrated that telomere length is highly variable from birth onwards with longer telomere in newborn girls compared with newborn boys. Telomere length shortens with aging [62] and additionally is under the influence of a combination of factors including genetic traits and environmental factors [63]. Among them, high levels of chronic psychological stress, obesity, smoking, extreme physical activity have been associated with increased oxidative stress and/or inflammation [15], two mechanisms which are known to accelerate telomere shortening [64–67].

Therefore, it is reasonable to assume that longer telomeres at birth result in a better cellular capacity to cope with oxidative and inflammatory conditions during the course of life [17, 28]. MiRNAs may be one of the hallmarks in the regulation of the aging process by controlling the function of telomeres [18]. Our current key finding suggests a sex-specific association of telomere length and candidate miRNA expression. In particular, higher placental expression of miR-34a, miR-146a, miR-210 and miR-222 is associated with longer placental telomere length at birth, in newborn girls. Taken together, each doubling of the studied placental miRNA expression was associated with longer placental telomere length within the range of 2.8–3.6% in newborn girls. In newborn boys, we found that each doubling in placental miR-21 was associated with 2.2% shorter telomere length. In this observational study, the significant miRNAs explain around 6–8% of the telomere length variance at birth which is considerable as the telomere length is a multifactorial trait.

A growing body of evidence illustrates the association between miRNA expression and maternal exposure to environmental stressors [38, 39, 42, 43, 68, 69]. Based on our previous work, most of these candidate placental miRNAs were found to be inversely associated with maternal pre-pregnancy BMI (miR-20a, miR-34a, miR-146a, miR-210 and miR-222) [39] and gestational exposure (2nd trimester) to particulate matter pollution (miR-20a, miR-21, miR-146a and miR-222) [38]. In another study, lower expression of miR-16, miR-21 and miR-146a has been reported in placentae from mothers who smoked during pregnancy [42]. These miRNAs play important roles in several key cellular processes (inflammation, oxidative stress, cell cycle, cellular senescence, apoptosis) which all have been described to be involved in aging process and seemingly in regulation of telomere length.

Notably, placental miRNA expression exhibits a sex-specific association with placental telomere length. This is consistent with our previous work, where only in newborn girls, placental miRNA expression (miR-20a, miR-34a, miR-146a, miR-210 and miR-222) was inversely associated with maternal pre-pregnancy BMI [39] (Fig. 1). We hypothesized that the identified decrease in placental miRNA expression with increasing maternal pre-pregnancy BMI, might be involved in placental inflammation and angiogenesis. Here, lower expression of these studied miRNAs was associated with shorter telomere length only in girls, as well. It might be that the placenta from newborn girls is more susceptible to maternal exposures to environmental stressors. In contrast, in newborn boys, the higher expression of miR-21 was associated with shorter telomere length. A plausible explanation may involve targeted genes by these miRNAs implicated in control of human telomerase reverse transcriptase (*hTERT*), which in turn may contribute to the sexual dimorphism on placental telomere length. Longer telomeres in women than in men have been attributed to upregulation of telomerase activity (in vitro) and inhibition of oxidative stress by estrogen and subsequent inhibition of telomere shortening [70], which might also account for the difference in placental telomere length. Nevertheless, it still remains unknown how these dysregulated miRNAs can affect this process in placenta in a sex-specific manner.

Our candidate miRNAs have been previously seen to be dysregulated during aging [71–73]. A genome-wide miRNA analysis in blood of persons above 90 years (n = 15) vs. middle-aged individuals (n = 55) revealed 80 significant differentially expressed miRNAs, with the majority (80%) to be downregulated in the persons at high age, among them miR-20a, miR-21 and miR-222 [71]. Decreased levels of miR-146a expression has been associated with aging in human umbilical vein endothelial cells (HUVEC) [72]. Downregulation of miR-16 and miR-34a have been also reported in peripheral blood mononuclear cells (PBMC) with human aging [73].

Shortening of telomere length occurs by each cell division, while elongation is partially regulated by telomerase activity [74]. *TERT* is needed for the maintenance of telomere length and plays the most critical role in telomerase activity [75]. The telomere length measured in an individual at any given point in time is always the result of a complex interplay between telomerase activity, the underlying genetic make-up of an individual and environmental and lifestyle factors that have their respective influence on (epi)genetic factors and telomerase activity. Little is known about the involvement of miRNAs in the regulation of telomere length [75]. MiR-34a inhibits sirtuin 1 (*SIRT1*), a positive regulator of telomere length which in turn regulates *TP53*, and has been indicated as pivotal player in aging [76]. Lower expression of miR-34a has been previously associated with longer telomere length in patients with gallbladder cancer by regulating DNA damage [77]. Upregulated miR-34a was identified in aged human hearts, involving a novel direct miR-34a target, Phosphatase 1 Nuclear Targeting Subunit (*PNUTS*) which reduced telomere shortening [78]. MiR-146a was up-regulated in senescent cells and inversely correlated with telomere length and TERT activity, when measured in angiogenic cells from chronic heart failure patients and healthy control subjects [79]. MiR-222 was negatively linked to telomere length in hepatocellular carcinoma and possibly regulated *hTERT* in breast cancer [80]. MiR-21, known as oncogenic miRNA, found to be inversely correlated with *hTERT* expression in hypertrophic scar fibroblasts [81].

In line with previous studies, downregulation of most of the studied placental miRNAs can be linked to aging reflected by shorter placental telomere length, in case of newborn girls. Slattery et al. [75], observed mostly (97%) a positive association of miRNAs with telomere length measured in 363 individuals, but none of them was included in our selected miRNA set. However, most of the candidate placental miRNAs were found to be inversely correlated with telomere length in senescent or cancerous cells in previous studies [77, 79–81]. These discrepancies might be due to different biology system, different age of the study population or implication of aging-related diseases, whereas we focus on healthy population of newborns.

This is to our knowledge the first large study which investigates potential associations between placental miRNA expression and telomere length at birth. In newborns, placental telomere length is on average 8 kb [82] and the annual telomere loss in adult leukocytes is between 32.2 and 45.5 bp [10]. A reduction of 2–3.5% in telomere length in association with some of our miRNA targets corresponds approximately to a reduction of 160–280 bp. This indicates that our effect-sizes of miRNA on telomere length are equivalent to a loss of approximately 5 years (based on telomere attritions of 32.2–45.5 bp per year [10]), which illustrates the public health significance of our molecular targets, as based on telomeric year equivalence in adulthood. Our study showed consistent associations between unadjusted and adjusted models, indicating no strong confounding for the adjusted variables on our association. However, possible limitations should also be reported. This study is limited by the measurement of only candidate miRNA genes previously shown to be expressed in placental tissue in response to maternal exposures to environmental stimuli. There might be other miRNAs of interest in respect to telomere length regulation. More research is needed to provide additional confirmation on these associations. Moreover, although differentially miRNA expression between first and term human trophoblastic cell lines from normal pregnancies has been reported [83, 84], in our study no data on either placental miRNA expression or placental telomere length at earlier stages of pregnancy are available, as we performed those measurements only in placental tissue collected at birth. As our data are observational in nature and reflect the variability at birth we do not have information during earlier stages and also not on the direction (whether telomere length influences miRNA expression or vice versa). Lastly, we adjusted our statistical models for a set of possible covariates including drivers of inflammation (air pollution exposure and pre-pregnancy BMI). These showed the robustness of our data, but we cannot exclude the chance of other unknown uncontrolled variables to influence our findings, therefore residual confounding may exist. In this regard we cannot totally exclude that pro-inflammatory factors both influence telomere length and miRNA expression without a causal relation between the miRNA expression and telomere length. Validation of these findings in an independent cohort study is an interesting topic for future research.

## Conclusions

We have shown that in a sex-specific manner placental telomere length is altered in association with placental miRNAs involved in various biological processes including inflammation, oxidative stress, cellular senescence, cell cycle and apoptosis in a targeted approach. The studied miRNA expression revealed in general stronger associations with telomere length in girls than in boys providing new targets to understand sex-specific telomere dynamics from early life onwards.

## Additional files

**Additional file 1.** Trimester-specific PM_2.5_ exposure (µg/m^3^) with mean, interquartile range (IQR), 10th (P10) and 90th (P90) percentiles are given, in newborn girls and boys.

**Additional file 2.** Adjusted *p-*values after FDR correction for unadjusted, adjusted and sensitivity analyses.

## Abbreviations

miRNA: microRNA
RTL: relative telomere length
CI: confidence interval
PM_2.5_: particulate matter (aerodynamic diameter of less than 2.5 μm)
NO_2_: nitrogen dioxide
BMI: body mass index
HUVEC: human umbilical vein endothelial cells
PBMC: peripheral blood mononuclear cells
*(h)TERT*: (human) telomerase reverse transcriptase
*TP53*: tumor protein 53
*SIRT1*: sirtuin 1
*PNUTS*: phosphatase 1 Nuclear Targeting Subunit
FDR: false discovery rate
TRF1/2: telomeric repeat binding factor 1/2
