# Supplementary material for: Sex-specific associations between telomere length and candidate miRNA expression in placenta
Source: J Transl Med. 2018 Sep 12;16:254. doi: 10.1186/s12967-018-1627-z (PMC6134555; doi:10.1186/s12967-018-1627-z)
Supplement: Supplementary file 1 — Additional file 1. Trimester-specific PM2.5 exposure (µg/m3) with mean, interquartile range (IQR), 10th (P10) and 90th (P90) percentiles are given, in newborn girls and boys. [file 12967_2018_1627_MOESM1_ESM.docx]

**Additional File**

**The following are the supplementary data related to this article:**

***Sex-specific associations between telomere length and candidate miRNA expression in placenta***

Maria Tsamou^1^, Dries S Martens^1^, Bianca Cox^1^, Narjes Madhloum^1^, Karen Vrijens^1^, Tim S Nawrot^1,2^

**Affiliations:**

^1^Center for Environmental Sciences, Hasselt University, 3500, Hasselt, Belgium;

^2^Department of Public Health, Environment & Health Unit, Leuven University (KU Leuven), 3000, Leuven, Belgium;

**Table of contents**

**Additional File 1.** Trimester-specific PM_2.5_ exposure (µg/m^3^) with mean, interquartile range (IQR), 10^th^ (P10) and 90^th^ (P90) percentiles are given, in newborn girls and boys…………………………………………..………………………….…………………………………………………………………………….

**Additional File 2.** Adjusted *P-*values after FDR correction for unadjusted, adjusted and sensitivity analyses..…………………………………………………………………………………………………………………………………………….

**Additional File 1.** Trimester-specific PM_2.5_ exposure (µg/m^3^) with mean, interquartile range (IQR), 10^th^ (P10) and 90^th^ (P90) percentiles are given, in newborn girls and boys.

| PM_2.5_ exposure | Mean | IQR | P10 | P90 |
| --- | --- | --- | --- | --- |
| *Girls (n=105)* | | | | |
| Trimester 1 | 13.83 | 7.47 | 8.51 | 22.84 |
| Trimester 2 | 14.28 | 8.21 | 8.51 | 22.62 |
| Trimester 3 | 14.57 | 9.36 | 8.18 | 23.92 |
| *Boys (n=98)* | | | | |
| Trimester 1 | 13.98 | 7.77 | 7.98 | 23.42 |
| Trimester 2 | 14.24 | 8.63 | 8.43 | 20.83 |
| Trimester 3 | 14.39 | 9.17 | 7.49 | 23.26 |
