# Supplementary material for: Sex-specific associations between telomere length and candidate miRNA expression in placenta
Source: J Transl Med. 2018 Sep 12;16:254. doi: 10.1186/s12967-018-1627-z (PMC6134555; doi:10.1186/s12967-018-1627-z)
Supplement: Supplementary file 2 — Additional file 2. Adjusted p-values after FDR correction for unadjusted, adjusted and sensitivity analyses. [file 12967_2018_1627_MOESM2_ESM.docx]

**Additional File 2.** Adjusted *P-*values after FDR correction for unadjusted, adjusted and sensitivity analyses.

| miRNAs | | *Unadjusted Analysis* | | *Main*  *Analysis^a^* | | *Sensitivity Analysis* | | | | | |
| --- | --- | --- | --- | --- | --- | --- | --- | --- | --- | --- | --- |
|  |  |  |  |  |  | **Model 1^b^** | | **Model 2^c^** | | **Model 3^d^** | |
|  |  | ***P-value*** | ***FDR*** | ***P-value*** | ***FDR*** | ***P-value*** | ***FDR*** | ***P-value*** | ***FDR*** | ***P-value*** | ***FDR*** |
| *Girls* | | | | | | | | | | | |
| miR-16 | 0.068 | | 0.079 | 0.188 | 0.21 | 0.11 | 0.133 | 0.18 | 0.20 | 0.10 | 0.12 |
| miR-20a | 0.065 | | 0.079 | 0.16 | 0.21 | 0.10 | 0.133 | 0.14 | 0.20 | 0.044 | 0.061 |
| miR-21 | 0.32 | | 0.32 | 0.52 | 0.52 | 0.27 | 0.272 | 0.51 | 0.51 | 0.33 | 0.33 |
| miR-34a | 0.012 | | 0.021 | 0.012 | 0.050 | 0.010 | 0.039 | 0.013 | 0.047 | 0.005 | 0.015 |
| miR-146a | 0.008 | | 0.021 | 0.016 | 0.050 | 0.017 | 0.039 | 0.015 | 0.047 | 0.006 | 0.015 |
| miR-210 | 0.010 | | 0.021 | 0.030 | 0.052 | 0.024 | 0.041 | 0.028 | 0.049 | 0.017 | 0.030 |
| miR-222 | 0.005 | | 0.021 | 0.022 | 0.050 | 0.016 | 0.039 | 0.020 | 0.047 | 0.004 | 0.015 |
| *Boys* | | | | | | | | | | | |
| miR-16 | 0.95 | | 0.95 | 0.44 | 0.98 | 0.20 | 0.57 | 0.38 | 0.75 | 0.35 | 0.57 |
| miR-20a | 0.73 | | 0.95 | 0.70 | 0.98 | 0.39 | 0.57 | 0.61 | 0.79 | 0.48 | 0.57 |
| miR-21 | 0.12 | | 0.84 | 0.085 | 0.59 | 0.043 | 0.30 | 0.082 | 0.57 | 0.061 | 0.43 |
| miR-34a | 0.64 | | 0.95 | 0.47 | 0.98 | 0.27 | 0.57 | 0.40 | 0.75 | 0.27 | 0.57 |
| miR-146a | 0.89 | | 0.95 | 0.84 | 0.98 | 0.71 | 0.71 | 0.75 | 0.79 | 0.49 | 0.57 |
| miR-210 | 0.94 | | 0.95 | 0.98 | 0.98 | 0.43 | 0.57 | 0.79 | 0.79 | 0.75 | 0.75 |
| miR-222 | 0.81 | | 0.95 | 0.60 | 0.98 | 0.49 | 0.57 | 0.43 | 0.75 | 0.25 | 0.57 |

*^a^ Main analysis was adjusted for newborn’s ethnicity, gestational age, paternal age, maternal smoking status, maternal educational status, parity, date of delivery and outdoor temperature during the 3^rd^ trimester of pregnancy (girls:n=105, boys:n=98).*

*^b^ Excluding newborns with non-European ethnicity (girls:n=98, boys:n=88).*

*^C^ Main analysis was additionally adjusted for pre-pregnancy BMI and gestational weight gain during pregnancy.*

*^d^ Main analysis was additionally adjusted for trimester-specific PM_2.5_ air pollution.*
